# Supplementary material for: Host range and zoonotic potential linked to P-like fimbrial (PLF) adhesin specificity in avian pathogenic Escherichia coli
Source: PLoS Pathog. 2026 Apr 6;22(4):e1013691. doi: 10.1371/journal.ppat.1013691 (PMC13068334; doi:10.1371/journal.ppat.1013691)
Supplement: S1 Fig — Luminescence from strain QT598 carrying the pPlf-lux reporter was measured over three consecutive passages in LB broth cultured without agitation (left) and with agitation (right) at 37°C. Under static conditions, luminescence remained stable across all passages with no significant difference (ns). Data represent mean ± SEM of biological replicates. (PDF) [file ppat.1013691.s001.pdf]

Supporting information

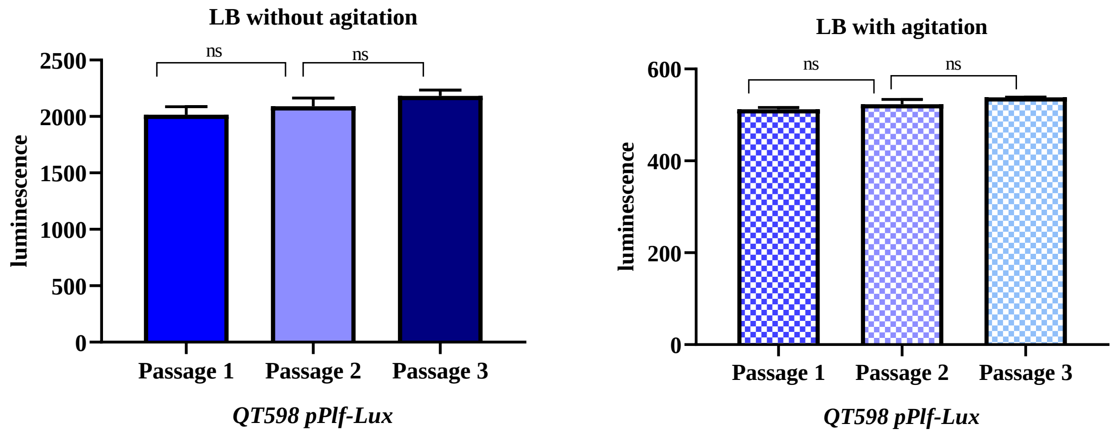

Fig S1. Stability of *plf* promoter activity across passages under different growth conditions.
