# Supplementary material for: Host range and zoonotic potential linked to P-like fimbrial (PLF) adhesin specificity in avian pathogenic Escherichia coli
Source: PLoS Pathog. 2026 Apr 6;22(4):e1013691. doi: 10.1371/journal.ppat.1013691 (PMC13068334; doi:10.1371/journal.ppat.1013691)
Supplement: S2 Fig — Luminescence from QT598 pPlf-lux was measured over three consecutive passages of growth on LB agar. The luminescence levels showed significant increases between passages. Data are the means from three independent experiments, and error bars represent standard errors of the means. *, P < 0.05; **, P < 0.01; and ***, P < 0.001, ****, P < 0.0001 using one-way ANOVA. (PDF) [file ppat.1013691.s002.pdf]

Supporting information

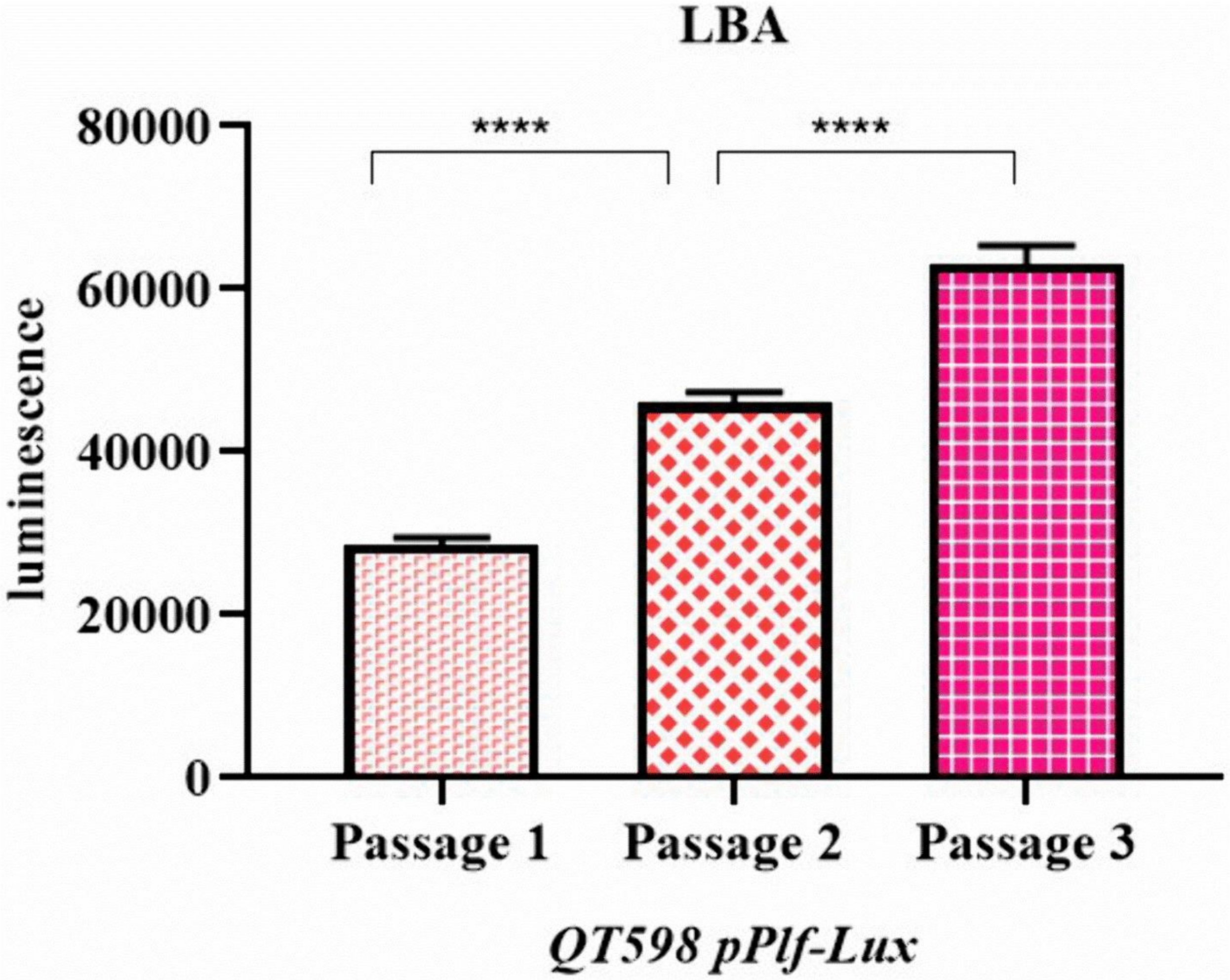

Fig S2. Increased Activity of *plf* promoter with consecutive passages on LB agar.
