# Supplementary material for: Host range and zoonotic potential linked to P-like fimbrial (PLF) adhesin specificity in avian pathogenic Escherichia coli
Source: PLoS Pathog. 2026 Apr 6;22(4):e1013691. doi: 10.1371/journal.ppat.1013691 (PMC13068334; doi:10.1371/journal.ppat.1013691)
Supplement: S3 Fig — Positive control is HA with strain QT4741 (PlfG class I adhesin cloned from strain UMEA-3703–1). Hemagglutination was not inhibited at concentrations ranging from 0.03 M to 0.1 M sodium metaperiodate, whereas blood was lysed at higher concentrations. Agglutination inhibition was visiualized after 30 min of incubation on ice. (PDF) [file ppat.1013691.s003.pdf]

Supporting information

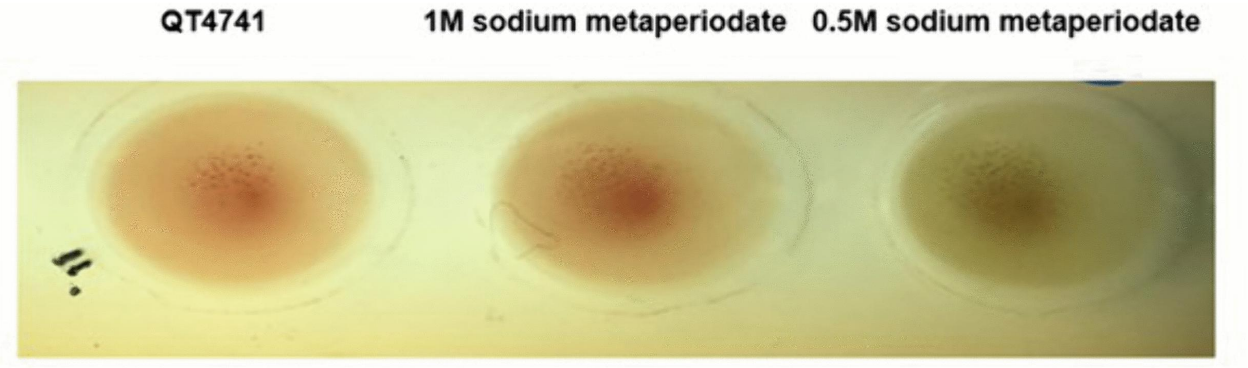

Fig S3. Macro-hemagglutination (HA) inhibition test of human O^{+} erythrocytes by PL fimbriae-producing strains in the presence of sodium metaperiodate.
