# Supplementary material for: Host range and zoonotic potential linked to P-like fimbrial (PLF) adhesin specificity in avian pathogenic Escherichia coli
Source: PLoS Pathog. 2026 Apr 6;22(4):e1013691. doi: 10.1371/journal.ppat.1013691 (PMC13068334; doi:10.1371/journal.ppat.1013691)
Supplement: S4 Fig — Positive control is HA with strain QT5726 (Clone PlfG class II from strain QT598), and negative control is QRN172. Hemagglutination was inhibited at concentrations ranging from 0.03 M to 0.1 M sodium metaperiodate, whereas blood was lysed at higher concentrations. Agglutination inhibition was visiualized after 30 min of incubation on ice. (PDF) [file ppat.1013691.s004.pdf]

Supporting information

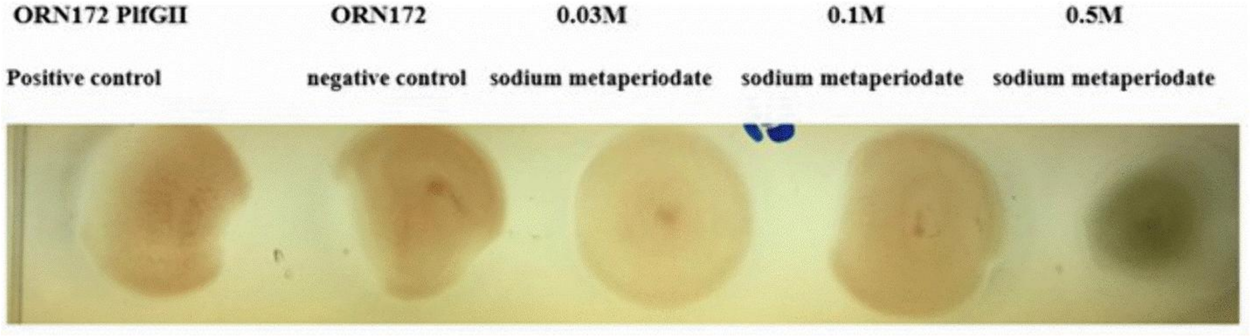

Fig S4. Macro-hemagglutination (HA) inhibition test of human O⁺ erythrocytes by PL fimbriae-producing strains in the presence of sodium metaperiodate.
