# Supplementary material for: Host range and zoonotic potential linked to P-like fimbrial (PLF) adhesin specificity in avian pathogenic Escherichia coli
Source: PLoS Pathog. 2026 Apr 6;22(4):e1013691. doi: 10.1371/journal.ppat.1013691 (PMC13068334; doi:10.1371/journal.ppat.1013691)
Supplement: S5 Fig — A) Positive control is HA with strain QT5726 (Clone PlfG class II from strain QT598), L-fucose and D-galactose were shown to inhibit HA by strain QT598, the negative control is QT598∆plf, and the other sugars can not inhibit the hemagglutination. B) Micro-hemagglutination inhibition test of turkey and human red blood cells. Positive control is HA with strain QT5726 (Clone PlfG class II from strain QT598), L-fucose and D-galactose were shown to inhibit HA by strain QT598, the negative control is QT598∆plf. Agglutination inhibition was visiualized after 30 min of incubation on ice. (PDF) [file ppat.1013691.s005.pdf]

Supporting information

A)

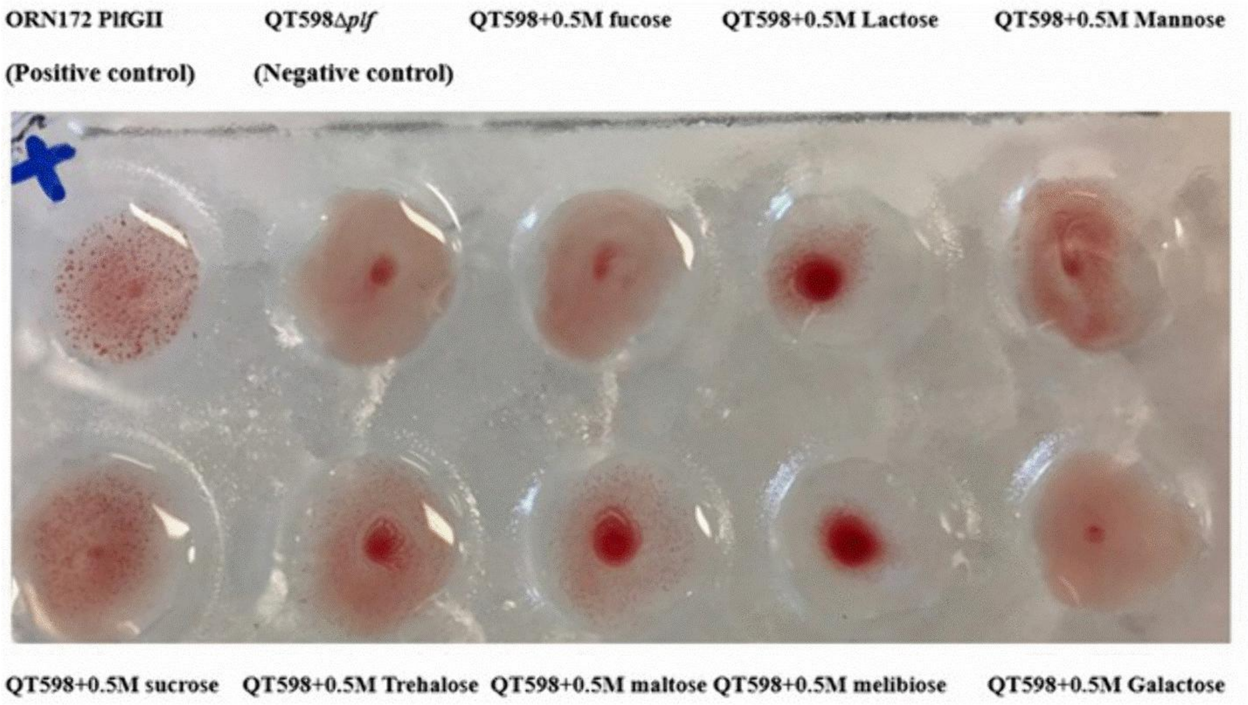

B)

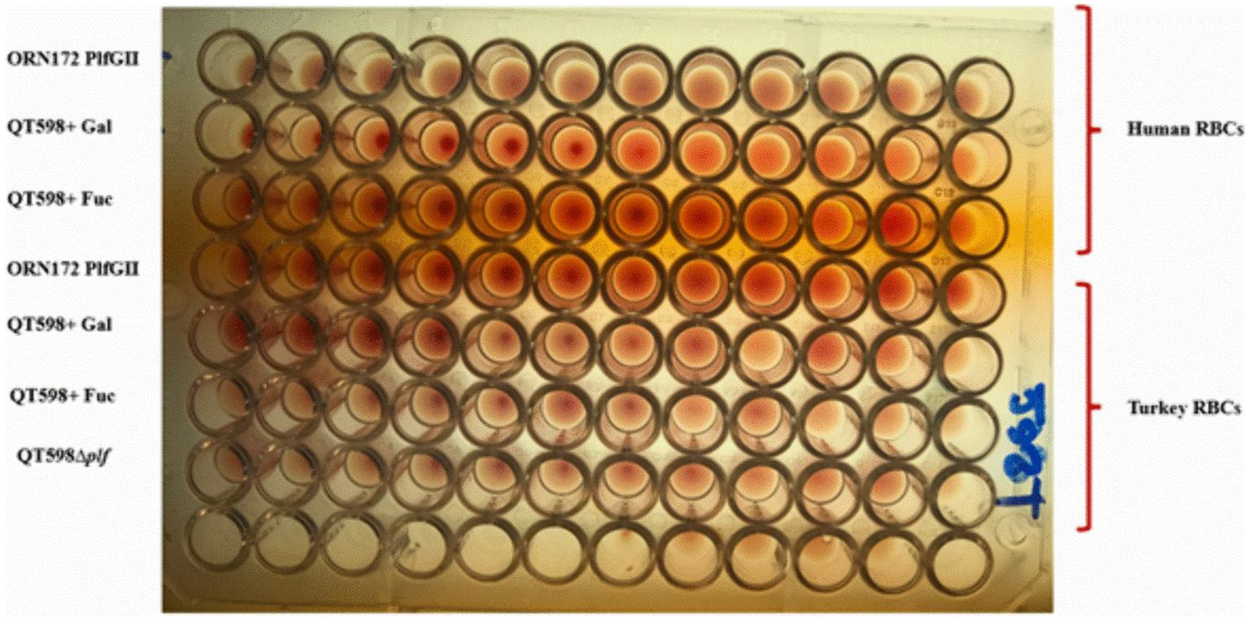

Fig S5. Macro-hemagglutination (HAI) inhibition and micro-hemagglutination inhibition tests of turkey and human erythrocytes by PL fimbriae-producing strains in the presence of various sugars. A) Macro-hemagglutination inhibition of QT598 in the presence of various sugars. Positive control is HIA with strain QT5726 (Clone PlfG class II from strain QT598) and negative control is QT598 Δ*plf* (QT4420). HIA assays showed that L-fucose and D-galactose inhibited hemagglutination at 15 mM, with effects observed at titers as low as 1:64 using turkey and human O^+ erythrocytes.
