# Supplementary material for: Host range and zoonotic potential linked to P-like fimbrial (PLF) adhesin specificity in avian pathogenic Escherichia coli
Source: PLoS Pathog. 2026 Apr 6;22(4):e1013691. doi: 10.1371/journal.ppat.1013691 (PMC13068334; doi:10.1371/journal.ppat.1013691)
Supplement: S6 Fig — Monolayers were infected for 2 h, and adherent bacteria were quantified by plating on LB Agar. Data are expressed as CFU/mL; bars represent means ± SEM from three independent experiments. QT5726 (ORN172 expressing PL fimbriae with PlfGII adhesin) ****P < 0.0001; ***P < 0.001; ns, not significant. (PDF) [file ppat.1013691.s006.pdf]

Supporting information

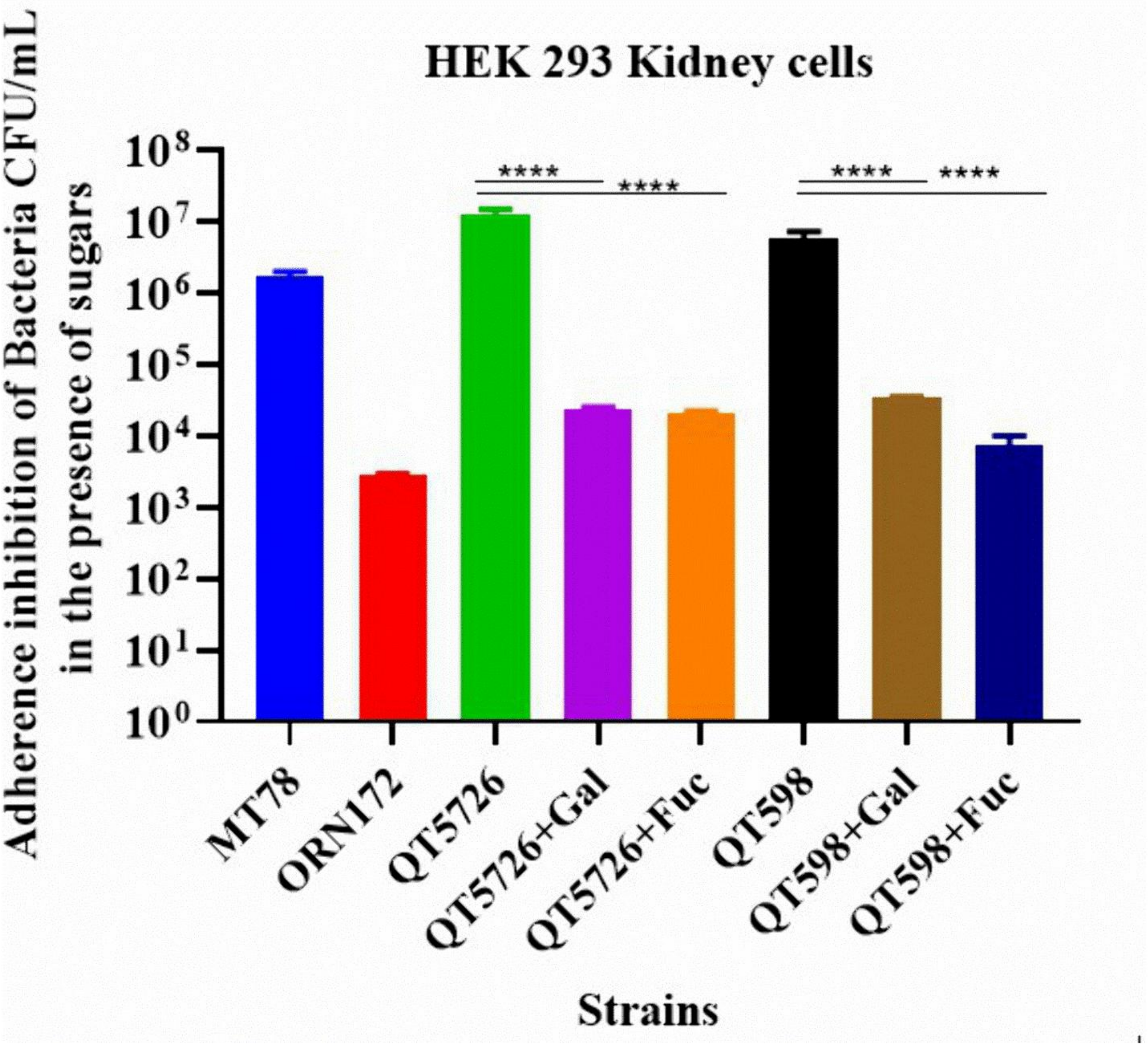

Fig S6. Adherence inhibition of Plf-expressing strains to HEK 293 kidney cells in the presence of inhibitor sugars.
