# Supplementary material for: Host range and zoonotic potential linked to P-like fimbrial (PLF) adhesin specificity in avian pathogenic Escherichia coli
Source: PLoS Pathog. 2026 Apr 6;22(4):e1013691. doi: 10.1371/journal.ppat.1013691 (PMC13068334; doi:10.1371/journal.ppat.1013691)
Supplement: S7 Fig — (A) Each slide is divided into four subarrays, with 100 distinct glycans immobilized on each subarray. (B) The table presents the spatial arrangement and numbering of glycans. For example, position 3 corresponds to mannose, position 61 to the H antigen, position 63 to the Lewis Y, and position 64 to the Lewis B. A complete list of glycans and their corresponding positions can be found on the RayBiotech Glycan Array 100 webpage. (PDF) [file ppat.1013691.s007.pdf]

Supporting information

A- The subarrays per glass chip

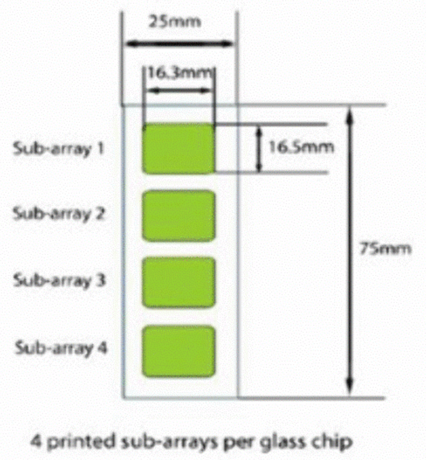

B- The numbering of glycans in the each sub-array

	1	2	3	4	5	6	7	8	9	10	11	12	13	14	15	16	17	18	19	20	21	22	23	24
1	POS1	POS1	POS1	POS1	POS2	POS2	POS2	POS2	POS3	POS3	POS3	POS3	NEG	NEG	NEG	NEG	1	1	1	1	2	2	2	2
2	3	3	3	3	4	4	4	4	5	5	5	5	6	6	6	6	7	7	7	7	8	8	8	8
3	9	9	9	9	10	10	10	10	11	11	11	11	12	12	12	12	13	13	13	13	14	14	14	14
4	15	15	15	15	16	16	16	16	17	17	17	17	18	18	18	18	19	19	19	19	20	20	20	20
5	21	21	21	21	22	22	22	22	23	23	23	23	24	24	24	24	25	25	25	25	26	26	26	26
6	27	27	27	27	28	28	28	28	29	29	29	29	30	30	30	30	31	31	31	31	32	32	32	32
7	33	33	33	33	34	34	34	34	35	35	35	35	36	36	36	36	37	37	37	37	38	38	38	38
8	39	39	39	39	40	40	40	40	41	41	41	41	42	42	42	42	43	43	43	43	44	44	44	44
9	45	45	45	45	46	46	46	46	47	47	47	47	48	48	48	48	49	49	49	49	50	50	50	50
10	51	51	51	51	52	52	52	52	53	53	53	53	54	54	54	54	55	55	55	55	56	56	56	56
11	57	57	57	57	58	58	58	58	59	59	59	59	60	60	60	[illegible]	61	61	61	61	62	62	62	62
12	63	63	63	[illegible]	64	64	64	64	65	65	65	65	66	66	66	66	67	67	67	67	68	68	68	68
13	69	69	69	69	70	70	70	70	71	71	71	71	72	72	72	72	73	73	73	73	74	74	74	74
14	75	75	75	75	76	76	76	76	77	77	77	77	78	78	78	78	79	79	79	79	80	80	80	80
15	81	81	81	81	82	82	82	82	83	83	83	83	84	84	84	84	85	85	85	85	86	86	86	86
16	87	87	87	87	88	88	88	88	89	89	89	89	90	90	90	90	91	91	91	91	92	92	92	92
17	93	93	93	93	94	94	94	94	95	95	95	95	96	96	96	96	97	97	97	97	98	98	98	98
18	99	99	99	99	100	100	100	100	NEG	NEG	NEG	NEG	POS3	POS3	POS3	POS3	POS2	POS2	POS2	POS2	POS1	POS1	POS1	POS1

Fig S7. Arrangement of glycans in each subarray of the glycan array slide.
