# Supplementary material for: Host range and zoonotic potential linked to P-like fimbrial (PLF) adhesin specificity in avian pathogenic Escherichia coli
Source: PLoS Pathog. 2026 Apr 6;22(4):e1013691. doi: 10.1371/journal.ppat.1013691 (PMC13068334; doi:10.1371/journal.ppat.1013691)
Supplement: S8 Fig — Positive control is strain QT5726 (ORN172 expressing PlfG class II cloned from strain QT598), negative control is ORN172. Addition of Lewis B or H antigen resulted in inhibiton of HA. Hemagglutination inhibition was scored visually after 30 min of incubation on ice. (PDF) [file ppat.1013691.s008.pdf]

Supporting information

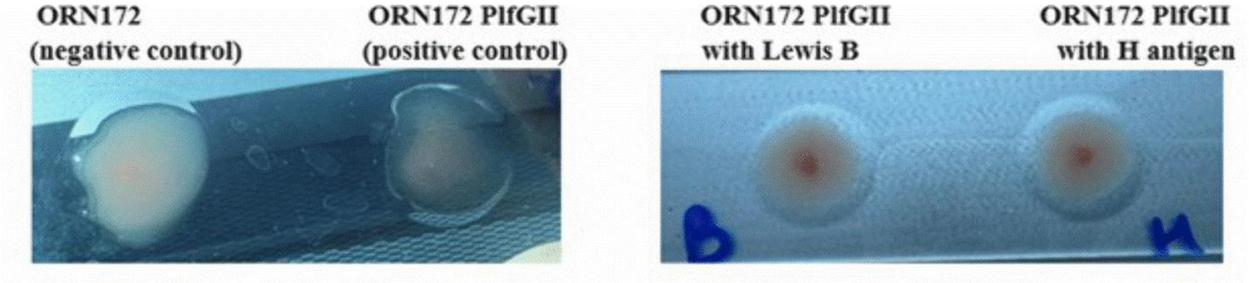

Fig S8. Hemagglutination inhibition (HAI) test of QT5726 with Lewis B antigen and H antigen with O+ RBCs.
