# Supplementary figures and images for: Host range and zoonotic potential linked to P-like fimbrial (PLF) adhesin specificity in avian pathogenic Escherichia coli
Source: PLoS Pathog. 2026 Apr 6;22(4):e1013691. doi: 10.1371/journal.ppat.1013691 (PMC13068334; doi:10.1371/journal.ppat.1013691)

# Supporting information

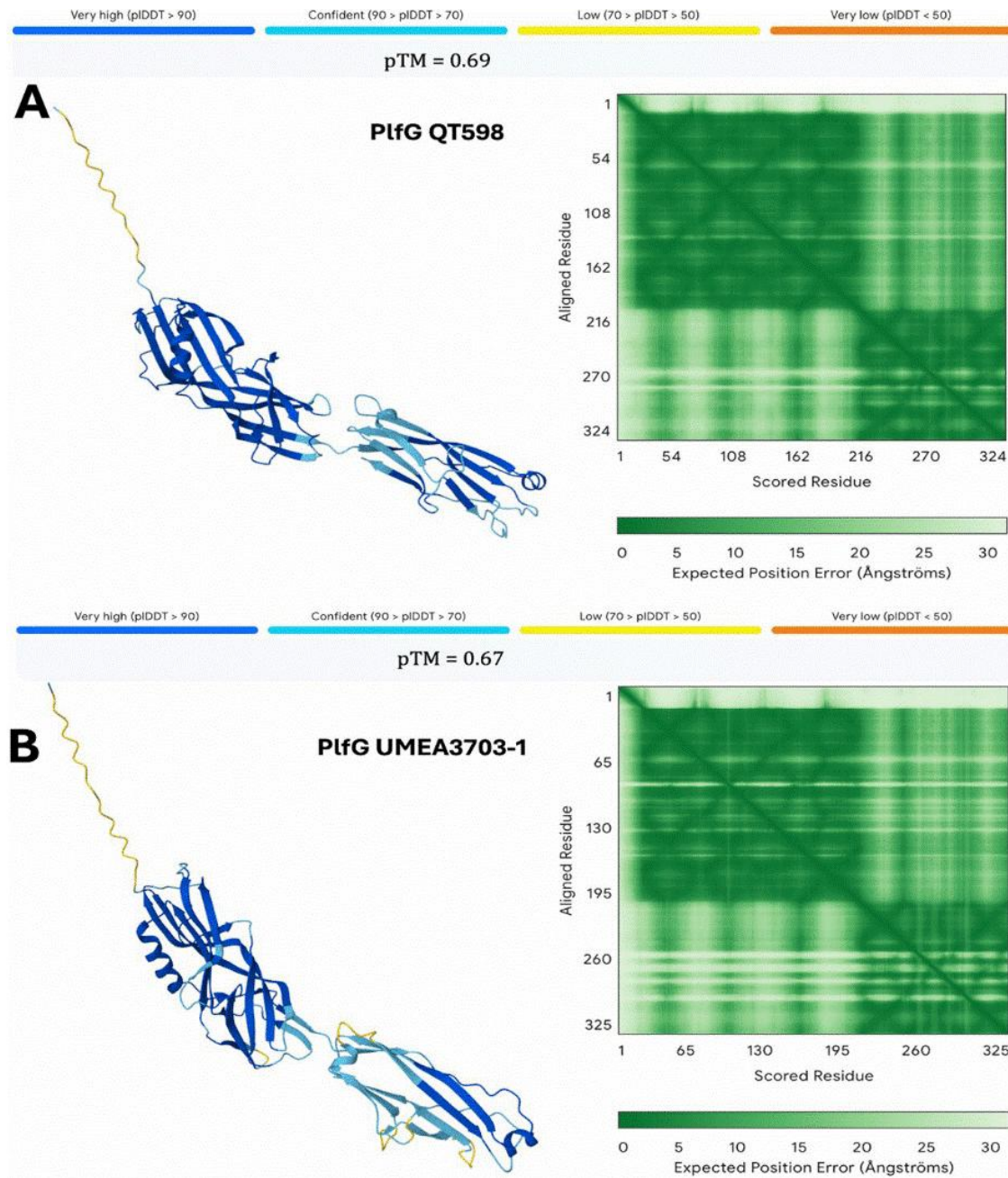

**Fig S9: AlphaFold model generation.**

Supplement: S9 Fig — The common amino acids are indicated in red between PlfG class II from QT598 and PlfG class I from UMEA 3703–1. (PDF) [file ppat.1013691.s009.pdf]

# Supporting information

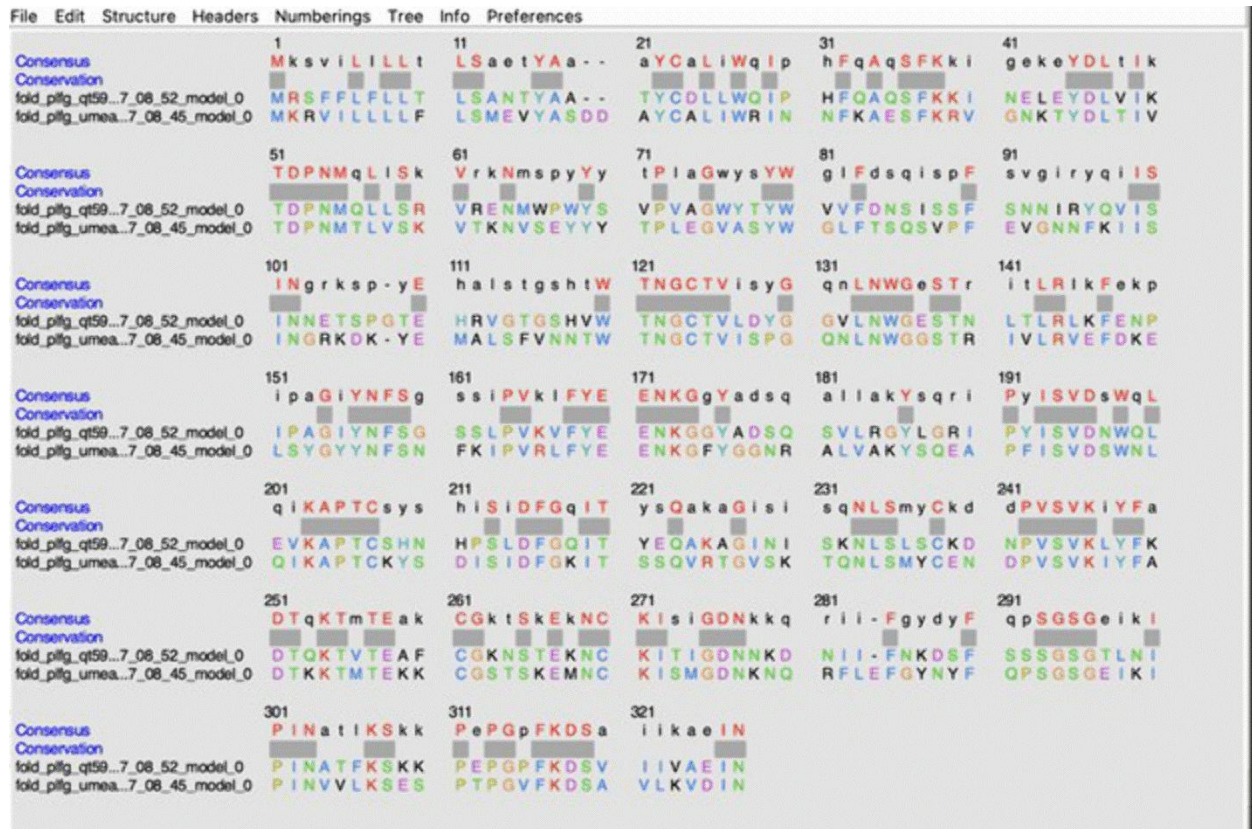

Fig S10. The amino acid alignment between PlfG class II and PlfG class I.

Supplement: S10 Fig — AlphaFold confidence metrics, including predicted aligned error (PAE), predicted local distance difference test (pLDDT), as well as and predicted TM-score (pTM) are shown for A) the PlfG QT598 and B) PlfG UMEA3703–1 models. (PDF) [file ppat.1013691.s010.pdf]
