# Supplementary material for: Host range and zoonotic potential linked to P-like fimbrial (PLF) adhesin specificity in avian pathogenic Escherichia coli
Source: PLoS Pathog. 2026 Apr 6;22(4):e1013691. doi: 10.1371/journal.ppat.1013691 (PMC13068334; doi:10.1371/journal.ppat.1013691)
Supplement: Attachment — Submitted filename: Responses to the reviewers PLOS PathogensAkrami.docx [file ppat.1013691.s012.docx]

**Response to the Reviewers’ Comments**

Manuscript ID: PPATHOGENS-D-25-02746

Title: Host range and zoonotic potential linked to P-like fimbrial adhesin specificity in avian pathogenic *Escherichia coli*

Journal: PLOS Pathogens

Dear Editor,

We would like to thank you and the reviewers for their careful evaluation of our manuscript and for their constructive comments. We have revised the manuscript accordingly, and all changes are highlighted in yellow in the revised marked version.

- **Part II – Major Issues: Key Experiments Required for Acceptance**
- **Reviewer #1**

**Comment:** No major issue.

- **Response:**
  *We thank the reviewer for their evaluation and confirm that no major revisions were requested.*
- **Reviewer #2**
- **Comment 1:**

The confocal images comparing adherence to turkey vs chicken lung sections rely on representative images and would benefit from quantitative analysis.

- **Response:**
  *We agree with the reviewer that quantitative support strengthens the interpretation of the confocal imaging data. We have now verified semi-quantitatively bacterial adherence by performing blinded counts of GFP-positive bacteria across multiple randomly selected fields per tissue section. These data have been incorporated into the revised Results section and are presented alongside the representative images to support the conclusion of species-specific tropism in the revised text now included on lines 259-262. Overall, adherence to turkey lung sections was 4-5X greater than to chicken lung sections.*
- **Comment 2:**

Clarification of glycan array specificity and whether binding is restricted to Fuc-α1,2-Gal–containing structures.

- **Response:**

*We thank the reviewer for emphasizing the importance and novelty of our glycan array data. No other glycans on the array showed comparable signals. The analysis supports the specificity of* ***PlfG class II adhesin*** *for these structures, consistent with inhibition by* ***L-fucose and D-galactose*** *observed in hemagglutination and tissue binding assays (Figs 5 and 7).*

*We included an image in which glycan spots showing meaningful binding are highlighted, demonstrating that the PlfG class II adhesin binds to Fuc-α1,2-Gal–containing Lewis b, Lewis y, and H antigens. Importantly, this image also shows that bacteria do not attach to other glycan spots, confirming the specificity of binding. Because the spots on the array are spatially separated, this presentation focuses on highlighting positive signals rather than providing a full spatial map of the array. (Numbers 1-3 spots are Geneticin and 4-6 spots are the sugar which is mentioned, as you see there is no binding between PL fimbriae and these sugars).*

- **Comment 3:**

Lack of direct demonstration that Lewis b/y or H antigens are present on turkey lung tissue.

- **Response:**
  *We agree with the reviewer that direct demonstration of Lewis b/y or H antigens on turkey lung sections is not provided in this study. Although, the specific types of glycans present on turkey cells and mucosal surfaces has not been confirmed, based on demonstrated binding-specificity to glycoarrays and inhibition with fucose and galactose, we can conclude that receptors recognized on turkey as well as human cells are likely to be glycans containing Fucose alpha-1, 2-Galactose structures at their surfaces. Despite not currently knowing any specific compositions of such glycans in avian species such as turkeys, the binding specificity strongly supports recognition of glycans with similar features to those of Lewis b/y and H antigens characterized from human cells and tissues.*
- **Comment 4:**

Clarification of HEK293 glycan background and sugar inhibition assay controls.

- **Response:**

*We thank the reviewer for this comment. We note that the glycan profile of HEK293 cells is not fully characterized with respect to fucosylated or galactosylated epitopes; therefore, regarding sugar concentrations and controls, we clarify that* ***D-galactose and L-fucose were used at 50 mM*** *in HEK293 adherence assays, consistent with concentrations applied in turkey tissue binding experiments to account for the more complex binding matrix (lines 357-359) revised manuscript). Prior to infection, bacteria were pre-incubated with sugars for 30 min at 4 °C (line 846 revised manuscript). In addition,* ***osmotic and toxicity effects were controlled*** *by including appropriate controls without sugars, as well as with sugars that do not affect PL fimbrial binding (e.g., glucose), which did not alter bacterial adherence, and we did not show this control data on the graph.*

- **Comment 5:**

AlphaFold-based structural modeling should be clearly presented as predictive.

- **Response:**

*We agree with the reviewer that the structural modeling data are predictive in nature. We have revised the text to explicitly state that the AlphaFold-based structural insights represent computational predictions rather than experimentally validated binding residues. We have also added a short paragraph outlining future mutagenesis and structural studies that could experimentally validate these predictions.*

- **Comment 6:**

Statistics and sample size clarification.

- **Response:**

*We thank the reviewer for emphasizing the importance of statistical transparency. We have revised all relevant figure legends (Fig 2) to clearly indicate the number of animals per group (n), the statistical tests used, and exact P values.*

*In addition, the Methods section now clarifies how data distribution was assessed and justifies the use of parametric or non-parametric tests for CFU data where appropriate.*

- ***Reviewer #3***
- ***Comment 1:***

*Lack of assays using tissue sections from different organs.*

- ***Response:***

*E. coli strain QT598 was originally isolated from a respiratory infection in a turkey poult, and the turkey air sac infection model was therefore selected as a biologically relevant host system to investigate the role of PL fimbriae during APEC infection. Using this model, we demonstrated that the wild-type strain efficiently colonizes the lungs, whereas deletion of the plf genes resulted in a significant reduction in pulmonary bacterial burdens. Restoration of lung colonization to wild-type levels upon genetic complementation provides strong evidence that PL fimbriae contribute directly to tissue-specific colonization in the avian host. To assess dissemination beyond the primary site of infection, bacterial burdens were also quantified in the liver and spleen. Reduced recovery of the plf mutant from these organs compared to the wild-type strain indicates a decreased capacity for systemic spread in the absence of PL fimbriae. No bacteria were detected in the bloodstream, suggesting that QT598 does not persist in circulation. Together, these data support a functional role for PL fimbriae in pulmonary colonization and limited dissemination to secondary organs, and justify the focus on lung tissue as the most relevant site for evaluating PL fimbrial function in this model.*

*We agree that the potential for fimbrial adhesins to also contribute to binding of other tissue sites in addition to the lung would also be of potential interest. PL fimbriae in addition to others such as type 1 fimbriae may contribute to colonization within the gut lumen, which may be an important source of meat contamination and foodborne transfer to humans. However, since the focus of our work was avian respiratory infection and tropism to human epithelial cells linked to urinary tract infections we did not investigate binding-specificity of the PL fimbrial adhesin to other tissues in poultry.*

- **Comment 2:**

Lack of qPCR expression data for the plf adhesin in different tissues.

- **Response:**

*Our decision to focus the qRT-PCR analyses on the air sac and lung was guided by both biological relevance and technical considerations. In the turkey air sac infection model, these tissues represent the primary sites of colonization and consistently yielded the highest bacterial burdens, allowing reliable recovery of bacterial RNA for quantitative analysis. In contrast, colonization of other organs such as the liver and spleen was sporadic and occurred in only a subset of birds, often at levels close to the detection threshold. Under these conditions, extracting sufficient, high-quality bacterial RNA for accurate gene expression measurements is technically challenging and may not faithfully reflect tissue-specific regulation. In addition, the absence of detectable bacteremia further supports the respiratory tract as the most relevant niche for examining plfA expression. For these reasons, we limited our analyses to respiratory tissues, where colonization was robust and reproducible, enabling meaningful interpretation of in vivo gene expression.*

- **Comment 3:**

Lack of quantitative data for hemagglutination and tissue section assays.

- **Response:**

*We agree that quantification strengthens these analyses. Semi-quantitative approaches to adherence assays lines 259-262 and HA experiments lines 310-312 are now described.*

- **Reviewer #4**
- **Comment:** No substantive concerns regarding the data or major conclusions.
- **Response:**

We thank the reviewer for the positive assessment of our manuscript and for recognizing the significance of our findings.

**Minor comments:**

Reviewer #1: General comments :

1- The names of the strains are different from one section and/or figure to another. Please harmonize for clarity.
   Example no 1: QT5726 (Fig S6) versus ORN172 PlfGII (Fig S5)
   Example no 2: QT5741(text L292) versus ORN172 PlfGI (Fig S3)

- *We thank the reviewer for pointing this out. We have carefully reviewed the manuscript and have revised all figures to harmonize the strain names throughout the text, figures, and supplementary material. Specifically, inconsistencies such as* ***QT5726 vs ORN172 PlfGII*** *and* ***QT5741 vs ORN172 PlfGI*** *have been corrected to ensure consistent and clear strain nomenclature across the manuscript.*

2- L160. Results for turkey erythrocytes could not be reviewed as they are not provided.

- *We apologize for this omission. The hemagglutination results obtained using turkey erythrocytes have now been added to the manuscript and are presented in the revised results section (line 155) and corresponding figure legend (lines 166-167). This addition allows direct comparison with human O⁺ erythrocytes and supports the conclusions drawn.*

3- L303-308. Please show the results of experiments with different concentrations of fucose and galactose.

- *We agree with the reviewer. The results obtained using a range of L-fucose and D-galactose concentrations have now been included. These data are presented in the revised Results section and shown in Figure S5B, demonstrating a concentration-dependent inhibition of hemagglutination.*

4- L530-533. It was not demonstrated that receptors of Plf were low or absent on chicken tissues or cells. Only reduced binding was shown. Please re-write.

*We agree that the sentence needed to be more accurate and have changed it to indicate that in contrast to the strong level of adherence conferred by PL fimbriae to turkey lung sections, a reduced or limited level of binding was observed with adherence assays using lungs sections from chickens. The text had been changed on lines 537-541.*

5- L113. Please add the year of isolation, disease caused by QT598, phylogenetic group and Sequence Type. Provide also a reference.

*QT598 is a phylogenetic group B2 strain isolated in 1995 from a 4-day-old turkey poult in France (a reference is also now provided). Lines 113-114*

6- L116. Could you please provide some details about the prevalence of plf genes in UPEC and APEC? Please provide also a reference.

*This information has been added lines 117-122.*

In a previous report first identifying and initially characterizing the *plf* gene cluster in 2022 ( reference ), we identified 686 distinct entries in the NCBI database that contained plf sequences. These entries were sourced from 171 human infections (mainly urinary isolates or blood infections) and 66 human fecal isolates. As well, strains encoding *plf* sequences were associated with infections in dogs, and samples from turkeys, chickens, and other avian species, and a variety of environmental sources.

Specific minor points :

L71. Delete “also”	*text was modified*
L77-79. This sentence is identical to that found in L48-50. *text was modified*
L113. Please add the year of isolation, disease caused by QT598, phylogenetic group and Sequence Type. Provide also a reference. *Information has been included*

L116. Could you please provide some details about the prevalence of plf genes in UPEC and APEC ? Please provide also a reference. *Information has been included*

L120. Fig S9 could be mentioned here. *Added to line 127*

L131. Please add “respectively”	*added on line 137*
L153. Please delete ‘with primers”. *This text was removed*
L181. CFU/ml of tissue. *Text modified*
L226 (and elsewhere). “in vitro” and “in vivo” should be italicized. *Done as requested*
L264. In A and B, please add “with turkey lung” (as in E and F) *text added lines 274-275*
L270. It would have been relevant to complement QT598 delta plf to confirm that binding to lung tissue was restored. *We verified western blot and MRHA which showed regain in PL fimbriae*
L276-277. Please indicate Class II for Plf in QT6131. *Text added lines 287-288*
L289. Please indicate the role of sodium metaperiodate. *Information was added* *lines 301-303*
L291. Delete “Fig S3” as no results from QT598. *Text modified lines 305-308*
L294. Please add “Fig S3”. *Text modified lines 305-308*
L297. Please indicate hemagglutination “of strain QT598”. *Text added to line 311*
L298. Please add “Fig 5A”. *added line 313*
L301. Please delete Fig S5
L303. Please delete "Fig 5A and S5" as these figures do not contain results of experiments with different concentrations of fucose and galactose. *Changes made as requested*
L341-343. This should be moved to Material and Methods. *Text removed and place in methods*
L343. MT78 was not found in the list of strains (Table 1). *The strain was now added*
L365. QT5320 was not found in the list of strains (Table 1). *It should be 5230, this was revised*
L373. “expressing PlfG classII” is already indicated L368. *Text modified*
L407. Black arrows can not be seen in Fig 7. *Text changed and figure to use “yellow circle”*
L479. Delete “also” . deleted line 527.
L671. Prior to homogenization, was BSG or any other solution added to tissue samples ? (which volume ?). *Text revised lines now on lines 690-691.*
L671. Cultures or tissues ? *Tissue homogenates* *line 691*
L704. Please provide plasmid name and source. *Name added at line 729, source listed in Table*
L717. “washing”	*word* corrected line 744.

- Response:

*We thank the reviewer for their careful reading of the manuscript and for the many helpful minor suggestions. We also updated references to supplementary figures, added missing clarifications in the Materials and Methods section, and revised figure annotations to improve clarity and visibility. Together, these revisions improve the readability and accuracy of the manuscript and fully address the reviewer’s minor comments.*

Reviewer #2:

1. Some typos and minor language issues throughout (e.g., “exression”, “moeities”, “PlgG” instead of PlfG, occasional double words like “also also”).

- *We thank the reviewer for noting these issues. The manuscript has been carefully proofread, and all typographical errors, spelling mistakes, and duplicated words have been corrected throughout the text.*

2. Ensure consistent terminology: PL fimbriae, PLF vs plf (gene cluster), PlfG (protein).

- *We agree with the reviewer. Terminology has been standardized throughout the manuscript to clearly distinguish between PL fimbriae, the* plf *gene cluster, and the PlfG adhesin protein.*

3. In the Methods:
The description of plasmids, strains, and primers is very detailed (Tables 1–2, pp. 28–31). This is fine, but a brief schematic overview of the mutant/complement construction (e.g., in a figure) might help readers.

*Thank you for this suggestion. The mutant construction and complementation strategy used in this study was straightforward and follows standard bacterial genetics procedures. We believe that the detailed descriptions provided in the Methods section and Tables 1–2 are sufficient for clarity and reproducibility. Therefore, we have not included any additional scheme or details to overview the mutagenesis and complementation experiments*

4. Check all abbreviations are defined at first use (e.g., MRHA, CFU/ml, LBA).

- *All abbreviations are now defined at their first occurrence in the text, and the manuscript has been reviewed to ensure consistent and clear usage throughout.*
- 5. For Fig 4 and Fig 5, specify magnification/scale bars and number of independent experiments quantified.
- *The figure legends for Fig 4 and Fig 5 have been revised to include this information.*

6. For Fig 7, make clear that each glycan is spotted in quadruplicate and that binding was observed in 3/4 spots, etc.

*This information was added to the figure 7 legend.*

7. Reference 10 requires correction, as the authors’ names are not included. Please provide the full citation details.

- *We apologize for this oversight. Reference 10 has been corrected to include the full list of authors and complete citation details.*

8. The Author Summary is essentially a duplication of the abstract. This section should be revised to communicate the significance of the findings in clear, non-technical language appropriate for the general public, rather than mirroring the scientific content of the abstract.

- *We agree with the reviewer. The Author Summary has been rewritten to avoid duplication of the abstract and to present the significance of the findings in clear, non-technical language appropriate for a general readership.*

Reviewer #3: Title: It does not represent the content of the study well in my opinion. The authors compare adhesion to chicken and turkey lung frozen sections, so they define host range within avian hosts, but I’m not sure how the zoonotic potential is investigated. It might be that everything becomes clear, when authors rewrite the results section.

*--- Thank you for your opinion. Since the adhesins also demonstrate a capacity to bind to human glycans and demonstrate adherence to specific human host epithelial cells such as bladder and kidney cells, this is why we have written that these PL fimbriae may contribute to tissue host specificity in the avian species, but also to zoonotic potential, as they clearly can mediate glycan-specific binding to human host cells linked to urinary tract infections.*

1- Line 39 Which tissue did you compared to show tissue-specificity of Plf class II?

*We have altered the text to read “avian species-specific tissue tropism”, as we agree we did not compare other types of tissues to compare specificity of adherence to different tissues or organs in this study.*

2- Line 48-50 - The zoonotic potential is not clear for me.

*See initial comment above.*

3- Line 133- 144 - Remove this section as it contains M&M and isn’t relevant to the manuscript.

*These results are important as the expression data allowed us to optimize in vitro expression of PL fimbriae for some of the expression and it was important as well as a baseline to compare the in vitro expression with in vivo expression from turkey respiratory tissues. We have decided to maintain this text.*

4- Line 145-162 - Remove this section (too much M&M) and mention the strain construction in 1-2 sentence in the next section. Fig. 1 – merge with Fig. 2. Provide bull blot image as supplementary data.

*As requested, we removed some of this information to the methods Section Instead of placing Fig.1 together with Fig. 2, since it is quite large, we opted to maintain the Western blot and HA data and the infection experiment results each as single figures.*

5- Line 197-199 – discussion. Is there possibility that this strain spreads through peritoneum not blood? Fig. 2 – did authors considered consulting statistician and discuss potential outlier removal?

*The normal route of dissemination of APEC strains is from the respiratory infection model(either tracheal or air sac) would typically be to the lungs from the air sacs and then potentially into the bloodstream with numbers increasing in the spleen and liver through this dissemination. Since this is not an intraperitoneal route, it is less likely that there could be spread from the peritoneum. We did not wish to remove outlier sampling and are aware that the animal infection models can result in some variation in levels of infection between animals. We nevertheless observed important statistical differences between the wild-type and mutant strain in our infection tests.*

6- Line 234-236 – Shouldn’t you compare expression of this fimbriae in different tissues/gut to make such a statement? Right now you have proof that host tissue induces expression of Plf

*We have altered the text to read “ that expression in the respiratory tract is increased compared to in vitro growth conditions and that the increased plf expression in the air sacs and lungs may contribute to the*

7- Line 248 – Please include 1 sentence to introduce the reader into the reason why this experiment was performed.

- *We thank the reviewer for this suggestion. A brief introductory sentence has now been added at the beginning of this section to clarify the rationale for the experiment, explaining that confocal microscopy was used to assess whether PL fimbriae contribute to host- and tissue-specific adherence to turkey versus chicken lung sections. Lines 252-255.*

8- Line 251 - “Many bacteria”, but where are quantitative data for results from this section?

- *We have now added semi quantitative data which demonstrated that adherence to turkey tissues was at least 4-5 X greater to turkey lung sections. Lines 265-268*

9- Fig. 4 - The use and organization of strains is not clear for me. Why not use wild type and Δplf in the analysis? Maybe also try to explain characteristics of the strains in figure caption? Please, add quantitative data for the results shown in these images.

- *The WT was used for the chicken and turkey sections. We have described some semi-quantitative data from analysis of microscope slides and bacterial cell counting. As described for point 8 above.*

10- Line 277 - “Lung tissue section from chicken, clone expressing Plf high-copy (QT6131).” A good example how clear figure caption is.

- *The caption is now corrected.*

11- Line 286-289 - Where are these results shown?

- *The pictures are provided in Figure 4. This is now specified Line 301.*

12- Line 289-290 – I would add few words to explain what metaperiodate treatment does to erythrocyte glycans.

*We have added to the text the fact that sodium metaperiodate treatment “, eliminates glycans from the cell surface, “ to lines 301-303.*

13- Line 291- Figure S3 and S4 are low quality

- *Better quality pictures have been added for the revised figures..*

14- Lines 292-294 – why you decide to use Plf class 1 in this experiment. It wasn’t use in the animal work.

- *The PlfG class I adhesin is quite distinct in sequence and phenotypic profile from the class II PlfG adhesin. We therefore also wished to investigate the potential sugar-binding specificity and discovered it likely binds targets other than sugars, since metaperiodate treatment does not abolish hemagglutination activity.*

15- Line 298-301 - Where are these results shown?

- *They are shown in figures S3 and S4. This was added to line 307.*

16- Line 302 – “HA” was that abbreviated earlier in the text?

- *Yes, it was abbreviated in the subtitle of the section.*

17- Line 303-305 - Where are these results shown?

- *They are shown in figures S3 and S4.*

18- Line 312-314 – please, move to M&M.

*We have placed this information in the methods*

19- Line 315-316 – Where is a bar plot with results? By how many precent was the attachment reduced?

*The addition of these sugars nearly completely eliminated the adherence to turkey sections based on visual inspection of microscope slides (90-95% inhibition). As there was almost complete loss of adherence, we did not generate any bar graphs for this data. A sentence was added indicating this strong level of inhibition (lines 328-329).*

20- Line 335 – Please include 1 sentence to introduce the reader into the reason why this experiment was performed. Why use HEK293? Why use Plf class I and II?

*We have added an explicative sentence here “ In a previous report, it was determined that PL fimbriae, which can be present in some avian pathogenic as well as uropathogenic E. coli from human infections could mediate adherence to urinary tract epithelial cells (reference Habouria paper)” Lines 348-350*

21- Line 341- 343 - please, move to M&M.

*As requested, we have moved this information to M&M.*

22- Fig. 6 - What is MT78?

- *It is an adherent APEC control strain known to adhere well to many cells by production of type 1 fimbriae and is now included in table 1.*

23- Line 358- 362 - please, move to M&M.

*Since the use of glycan arrays for this kind of study is not routine, we feel it is important to maintain the introduction of the protocol used in order to validate and confirm the results we obtained.*

24- Line 364-367- I would move positive control to supplementary data. Also, reduce the text describing positive control in the text.

*Inclusion of the controls is very important for the validation, as we were not certain we would be able to obtain valid results using fimbriae expressed on whole bacterial cells and we had no means of knowing what glycan specificity the PlfG adhesins might recognize. The use of type 1 (Fim) as a control was, as such, critical to confirm the assay and should be maintained as part of the main body of the results.*

25- Fig. 7. Why not show the whole array in the figure?

- ***Because individual glycans on the full glycan array slide cannot be clearly resolved without magnification, it is not feasible to present the entire slide in a meaningful way. For this reason, we used confocal microscopy to visualize bacterial attachment at higher resolution and to more accurately assess binding patterns.***

26- Line 415 - 417 - please, move to M&M.

*Text removed as requested.*

27- Line 417- 424 – Is that discussion or do you have results for this part?

- *This is a description of results presented in figure 8 data.*

28- Line 475 - 476- are these fimbriae plasmid or chromosome encoded?

- *Pap fimbriae are typically encoded on the chromosome.*

29- Line 481- 493- How this paragraph discusses results from this manuscript?

*It is a discussion to provide background of what is known or has been determined about this group of fimbriae and some strains that may produce them. As such, we think it is pertinent to include as part of the discussion..*

31- Line 499 - Have you tested adherence to other types of tissues?

*Changed text to “lectin-specific adherence to target tissues in the avian respiratory tract” . Lines 513-514*

*We have some preliminary data concerning adherence to trachea and air sacs, but have not verified other tissues. To avoid overstating, we have changed the text.*

32- Line 501 – “local tissue colonisation” – Shouldn’t the mutant be then more systemically spread. I think that this is associated with the model selected for the study.

*It is unclear whether the plf mutant may be more readily cleared from the lungs, but in either case loss of the fimbriae also certainly did not increase systemic spread to blood and other visceral organs.*

33- Line 511 - Citation for this statement would be helpful

*This is comparatively described within the references 12 and 30.*

34- Line 536 – Did you find them in human E. coli isolates?

*Yes, and this is why we have included additional information concerning distribution of this fimbriae among some APEC and UPEC strains to the text and also why we indicate there is also a zoonotic potential linked to the adherence profile of these fimbriae.*

35- Line 556-557 – On which figure have you shown these results?

*This was shown in Fig. 8 images.*

36- Line 575 – citation 50, it that correct one?

*Thank you for noticing this error. The correct reference should be the reference of Habouria et al (reference 19) in the original manuscript.*

Reviewer #4: Comment (minor)

Fig 3 and Fig 6. Data points for independent experimental replicates should be clearly shown as dots, rather than hidden by bars.

*As requested, we have modified these figures to individual dots instead of bar graphs.*
